# Effectiveness of Warm Compression at the Lumbosacral Region on Labor Pain Intensity During the First Stage of Labor Among Parturient Women: A Randomized Controlled Trial

**DOI:** 10.7759/cureus.115836

**Published:** 2026-09-05

**Authors:** Sharmistha Patel, Lily Podder, Geeta Bhardwaj, Ajay Halder

**Affiliations:** 1 School of Medico-Legal Studies, National Forensic Sciences University, Gandhinagar, IND; 2 College of Nursing, All India Institute of Medical Sciences, Bhopal, Bhopal, IND; 3 Obstetrics and Gynaecology, Bansal Hospital Bhopal, Bhopal, IND

**Keywords:** active stage, first stage of labor, heat therapy, intensity of labor pain, non-pharmacological method for labor pain, parturient women, warm compression

## Abstract

Introduction: Labor pain is a fundamental, complex, and subjective experience during childbirth, characterized by severe discomfort that can be difficult for many women to tolerate. There are many non-pharmacological therapies available for the relief of labor pain, such as aromatherapy, music therapy, acupressure, yoga, breathing exercises, etc. The present study was undertaken to assess the effectiveness of warm compression at the lumbosacral region on the intensity of labor pain during the first stage of labor.

Methods: A randomized controlled trial was conducted at the labor room of a tertiary care hospital in central India from October to November 2023 among 50 parturient women without any pregnancy-related complications, recruited through a convenience sampling technique and allocated randomly into interventional (n = 25) and control (n = 25) groups using sequentially numbered opaque sealed envelopes (SNOSE). The interventional group received warm compression at the lumbosacral region (L5-S1), given twice for a duration of 20 minutes with an interval of 30 minutes in the active stage of labor, whereas the control group received routine obstetrical care. The effectiveness of the intervention was measured by assessing the pain intensity before and immediately after the firstand second warm compression and 30 minutes after the second warm compression using the visual analogue scale (VAS). The collected data were analyzed using IBM SPSS Statistics for Windows, Version 24 (Released 2016; IBM Corp., Armonk, New York, United States).

Results: Both groups were homogeneous in terms of sociodemographic and clinical characteristics (p > 0.05). No significant difference was found between the intervention and control groups before and immediately after the first warm compression (p > 0.05). However, a significant difference was found immediately and after 30 minutes of the second warm compression (p < 0.05).

Conclusions: Warm compression is an easy, effective, and midwife-led method to reduce pain and enhance comfort in the first stage, helping women stay relaxed and participate actively in childbirth. It promotes caregiver support and enhances overall satisfaction with the birthing experience.

## Introduction

Childbirth encompasses many aspects: physical, emotional, psychological, social, and cultural [1]. It is a natural and spontaneous process that has occurred physiologically for many years [2]. The process of childbirth comprises three stages: the first stage starts from true labor pain to full dilatation of the cervix; the first stage is divided into three phases (latent, active, and transition). In this phase, labor contractions become more painful and increase in frequency and intensity [3]. Childbirth is frequently accompanied by significant pain, with studies indicating that approximately 60-70% of women experience severe pain during labor [2]. Labor pain is a natural and inevitable component of the childbirth process, experienced by nearly all women. It is often described as one of the most intense forms of pain in a woman’s life; however, its perception and tolerance vary greatly among individuals, influenced by physiological, psychological, and sociocultural factors [2,4]. Labor pain encompasses two main components: visceral and somatic. Understanding labor pain anatomy is crucial for effective pain management. Visceral pain, occurring in early and later stages, originates from cervical stretching and involves lower abdominal and pelvic nerves. Somatic pain emerges later, continuing into the second stage, from vaginal, cervical, and perineal nerves triggered by stretching and injury [5]. Fear and anxiety are closely associated with heightened perception of labor pain, often intensifying its severity. This emotional distress can make the pain extremely difficult for women to tolerate, thereby affecting their ability to cope effectively during childbirth [6]. The fear of normal labor pain contributes to approximately 59% of cesarean section cases [7]. According to data from the fifth phase of the National Family Health Survey (NFHS-5), the cesarean section rate in India was 14.3% in the public sector and 47.3% in the private sector [8].

Labor pain can be managed through two approaches: pharmacological and non-pharmacological methods [9]. Pharmacological methods, favored in high-income and developing countries, are standard due to their pain-reducing effectiveness. It also carries risks, such as epidurals causing hypotension, fever, and an increased likelihood of requiring assisted delivery [10]. About 90% of parturient women are satisfied with non-pharmacological methods. These include aromatherapy, music therapy, cold compression, heat therapy, acupressure, yoga, and breathing exercises [11]. Non-pharmacological methods of labor pain management are widely recognized for their safety and supportive role in the childbirth process; unlike pharmacological approaches, these do not interfere with the physiological progression of labor, uterine contractility, or fetal well-being. They are free from drug-related adverse effects such as maternal sedation, respiratory depression, or neonatal complications [6]. However, adopting non-pharmacological pain management (NPPM) methods faces obstacles like limited availability, lack of understanding, and cultural expectations. Overcoming these requires targeted approaches such as improving healthcare infrastructure, increasing education, aligning with cultural norms, and customizing information to individual choices. Additionally, expansive, standardized research studies are crucial for understanding the efficacy and long-term implications of NPPM techniques [12]. A notable finding was noted in the study, which was carried out by Durmuş and Eryilmaz, where the massage and hot-pack applications reduced perceptions of pain in the active and transition phases of labor, where the mean Visual Analogue Scale (VAS) scores of the massage and hot-pack application groups were lower than those of the control group (p < 0.001) [13]. Heat therapy as a pain management technique during labor functions by stimulating thermoreceptors in the skin and deeper tissues. This stimulation generates sensory impulses that modulate pain transmission at the spinal cord level through the “gate control theory” mechanism, thereby inhibiting the propagation of pain signals to the brain and reducing the perception of pain. Furthermore, the application of warm compresses facilitates vasodilation within the vascular system, enhancing blood circulation to the pelvic muscles. Improved perfusion aids in muscle relaxation, decreases uterine ischemia, and contributes to overall maternal comfort. Additionally, the soothing effect of heat therapy helps alleviate anxiety and fear, promoting psychological well-being and a more positive labor experience [11,14]. Several studies have demonstrated that the application of warm compression provides immediate relief from labor pain, particularly in the lumbosacral region; however, further research is warranted to evaluate its sustained effectiveness. Previous studies have utilized various heat application methods such as pelvic belts, heating pads, and hot water bags, yet these devices may not be readily available in all clinical or resource-limited settings. Therefore, the present study explores the use of cotton cloth as a warm compression medium, offering a simple, affordable, and accessible alternative for pain relief during labor, especially in rural and low-resource healthcare settings.

## Materials and methods

Study design and participants

A two-arm parallel-design prospective randomized controlled trial (RCT) was conducted among parturient women admitted to the labor unit of the Department of Obstetrics and Gynaecology, All India Institute of Medical Sciences (AIIMS), Bhopal, India. AIIMS Bhopal is an Institute of National Importance established by the Government of India and functions as a tertiary care teaching and referral hospital providing comprehensive maternal and neonatal healthcare services. The labor unit manages approximately 100-150 normal vaginal deliveries per month, serves as a referral centre for women from both urban and rural areas, and provides care for both low-risk and high-risk pregnancies. Routine intrapartum care followed standardized institutional protocols, including maternal and fetal monitoring, assessment of labor progress using a partograph, and supportive nursing care. Warm compression was administered as an adjunct to routine obstetric care. Pharmacological labor analgesia was not routinely provided; therefore, non-pharmacological measures were commonly used to promote maternal comfort during labor. The findings should be interpreted within this clinical context, as the availability of labor analgesia and standard maternity care practices may vary across healthcare settings. The participants were divided into two groups: a control group that received routine obstetrical care and an intervention group that received warm compressions. Informed consent was obtained from all participants after providing detailed information about the study.

A normal course of pregnancy and labor was defined as an uncomplicated singleton pregnancy at term with a live fetus in vertex presentation, spontaneous onset of labor, no undue prolongation of labor, and absence of maternal or fetal complications affecting the health of the mother or baby. Women who were in the active phase of the first stage of labor, with cervical dilatation ranging from 4 cm to full cervical dilatation, were included in the study. Women were excluded from the trial if they had a bedsore in the lumbosacral region, had undergone spinal surgery, or had maternal hypotension, as the potential vasodilatory effects of warm compression could further reduce blood pressure.

Ethical approval was obtained from the Institutional Human Ethics Committee of AIIMS, Bhopal. The study was registered with the Clinical Trials Registry-India (CTRI) (CTRI/2023/10/0584; LOP No. AIIMS/BPL/IHECSR/July/22/MscN/18). The study was conducted from 10 October 2023 to 1 November 2023 after obtaining approval from the Institutional Ethics Committee.

Sampling size and randomization

The sample size was calculated using OpenEpi software (Dean AG, Sullivan KM, Soe MM. OpenEpi: Open Source Epidemiologic Statistics for Public Health) based on a previous RCT by Kaur et al. [15], which involved nullipara women and evaluated the effectiveness of a warm compression on the lumbosacral region in terms of labour pain. That study reported a mean difference of 1.3 in labor pain between intervention and control groups. Assuming 80% power, a 5% significance level, and anticipating up to 10% attrition, a minimum of 25 participants per group was estimated. Eligible parturient women who met the inclusion criteria were recruited using a convenience sampling technique. After confirming eligibility, obtaining written informed consent, and completing the baseline assessment, participants were randomly assigned in a 1:1 ratio to the experimental or control group using a computer-generated simple randomization sequence. The computer-generated randomization sequence and numbered, opaque, sealed envelopes (SNOSE) were prepared by a postgraduate classmate who was not involved in participant recruitment, intervention delivery, outcome assessment, or data analysis. After enrolment and completion of the baseline assessment, the nursing officer attending the labour room provided the next numbered, opaque, sealed envelope to each eligible participant. The envelope was opened only at that stage to reveal the group allocation, and the participant was assigned to either the experimental or control group according to the allocation indicated in the envelope. This procedure ensured allocation concealment until enrolment and completion of the baseline assessment.

Data collection instruments

The data collection tool used in this study included a demographic sheet, which is self-structured, containing four items: age, education, occupation, and BMI. The clinical sheet, which is self-structured, comprised six items: gravida, participation in antenatal exercise, previous mode of delivery, number of abortions, nature of labour, onset of labour, and any previous medical conditions. Pain intensity was assessed using the VAS, a standardized and open-access tool used to measure pain intensity [16]. It consists of a 100 mm horizontal line, anchored by two descriptors at each end: “no pain” (0 mm) on the left and “worst imaginable pain” (100 mm) on the right. The scores were categorized as no pain (0-4 mm), mild pain (5-44 mm), moderate pain (45-74 mm), and severe pain (75-100 mm). The validity of the demographic and clinical data collection sheets was established, with validity indices of 0.92 and 0.91, respectively. Inter-rater reliability was assessed by two raters and demonstrated high reliability (r = 0.93).

Figure 1 shows the flow of participants through the study, including recruitment, allocation, follow-up, and analysis, and is illustrated according to the Consolidated Standards of Reporting Trials (CONSORT) Statement guidelines [17].

**Figure 1:**
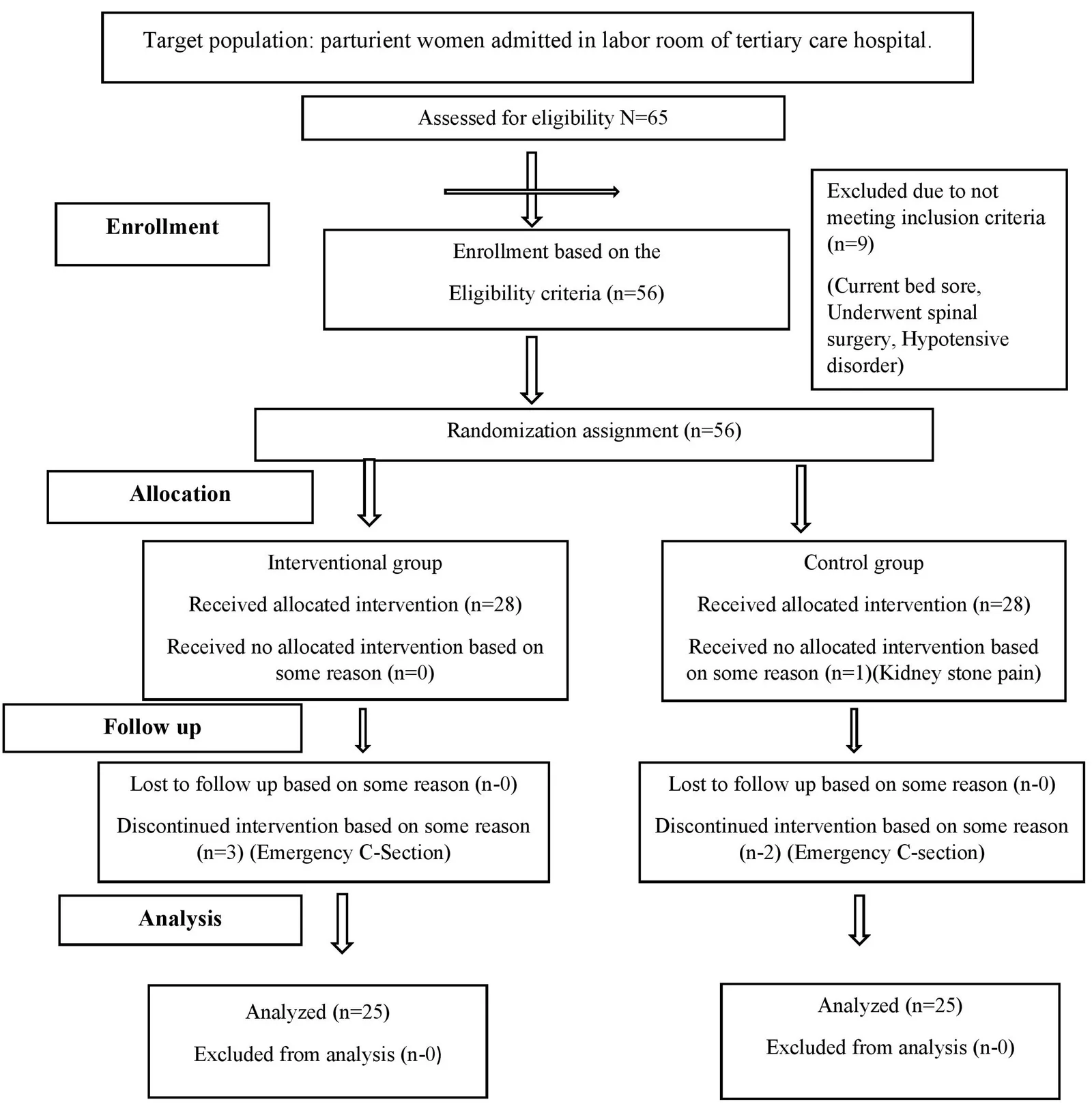
CONSORT flow diagram of the study CONSORT: Consolidated Standards of Reporting Trials

Intervention

In this study, participants in the intervention group received warm compression at the lumbosacral region (L5-S1), which was selected because it is a common site of referred pain during labor. Pain arising from uterine contractions and cervical dilatation is transmitted through visceral afferent fibers (T10-L1) and frequently radiates to the lower back as labor progresses [5]. Warm compression was administered using a pure cotton cloth (15 cm × 15 cm × 1 cm) dipped in warm water maintained at 50-60°C [15]. The warm compress consisted of a folded 100% cotton cloth that served as the interface between the warm water and the participant’s skin; no additional protective layer was used. The temperature of the compress was verified using a calibrated lotion thermometer before each application. Throughout the intervention, participants were continuously observed for signs of discomfort or adverse skin reactions, including excessive warmth, erythema, or burns. Participants were informed that they could request temporary interruption or removal of the warm compress at any time if they experienced discomfort. No adverse events or skin injuries were observed during the intervention period. The intervention was provided by the researcher. It was given twice for 20 minutes (mins) duration with 30 mins interval in the active phase of labor. Every five mins, the cloth was replaced with another warm cloth to maintain consistency in the temperature of the warm compression; hence, the cloth was replaced thrice during each 20 mins duration of the warm compression. The control group received routine obstetrical care as per the institutional policy. Pre-test and post-test intensity of labour pain in both groups was assessed using the VAS [16]. Post-test intensity of labour pain was assessed in the intervention group immediately after the first and second compression and after 30 mins following the second warm compression.

Figure 2 shows the detailed intervention protocol implemented during the study.

**Figure 2:**
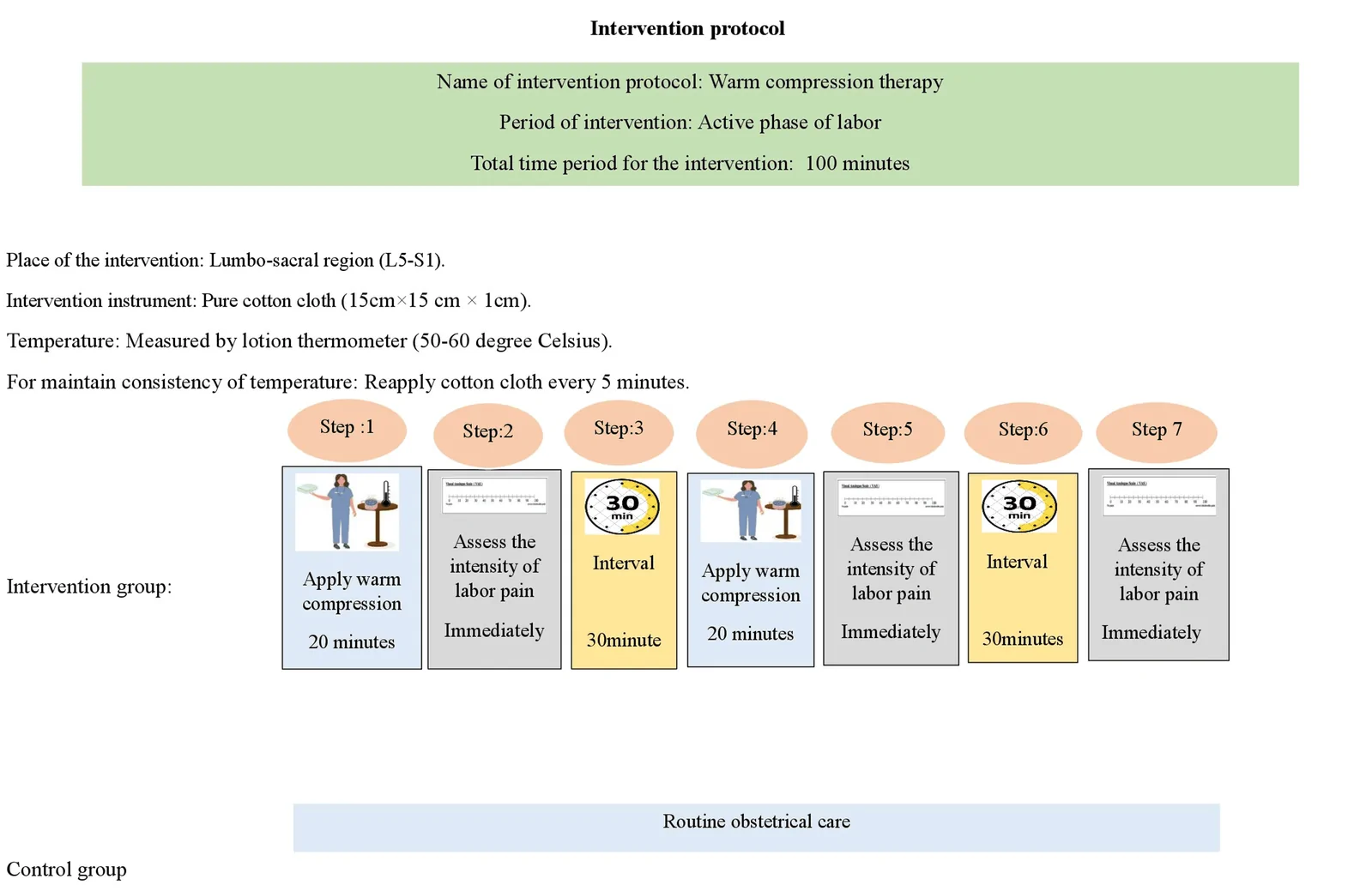
Intervention protocol The image was created by the authors using Canva (Canva Pty Ltd., Sydney, New South Wales, Australia).

Statistical analysis was conducted using IBM SPSS Statistics for Windows, Version 24 (Released 2016; IBM Corp., Armonk, New York, United States). The normality of the data was assessed using the Kolmogorov-Smirnov (K-S) test. Depending on the distribution, appropriate parametric or non-parametric tests were considered. A two-way repeated measures ANOVA (RM-ANOVA) followed by Bonferroni post hoc comparisons was applied to examine the effectiveness of warm compression at the lumbosacral region on intensity of labor pain across different time points. Associations between intensity of labor pain and selected demographic and clinical variables were evaluated using the chi-square test or Fisher’s exact test, as appropriate. A p-value < 0.05 was considered statistically significant. Figure 3 shows the timeline of the intervention and data collection procedures conducted during the study.

**Figure 3:**
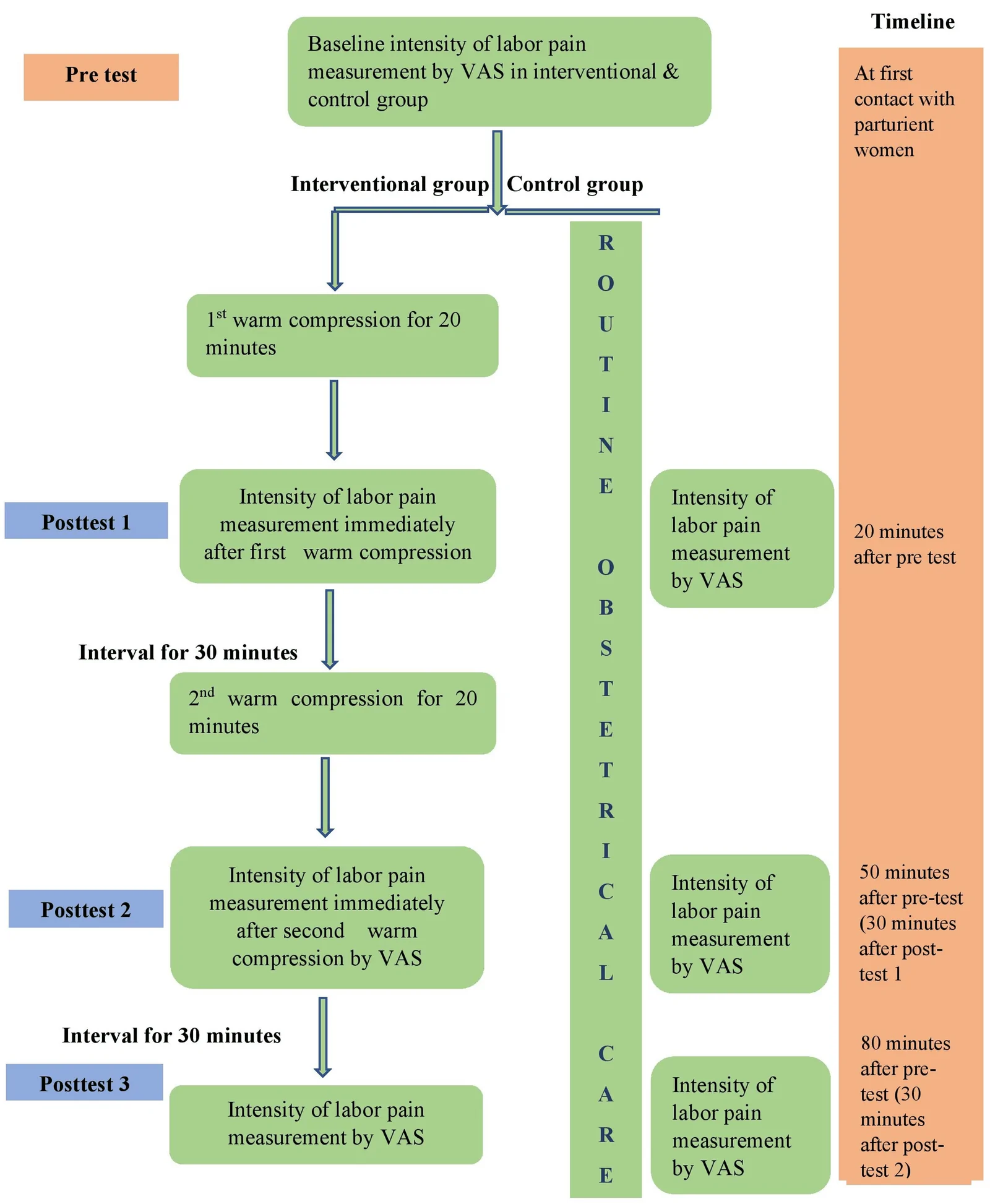
Timeline of intervention VAS: Visual Analogue Scale

## Results

The Kolmogorov-Smirnov test confirmed that the data were normally distributed (p > 0.05). Therefore, parametric analyses were applied for subsequent comparisons. With respect to baseline characteristics, the study groups were comparable. Homogeneity testing showed that there were no statistically significant differences between the intervention and control groups across demographic and clinical variables (p > 0.05), confirming that the groups were homogeneous at baseline. Among the participants, 44.0% of the intervention group and 52.0% of the control group were within the 25-30 years age range. The majority of parturient women in the control group (80%) and intervention group (56.0%) had normal BMI. Although a slight baseline variation in BMI distribution was observed, this difference was not statistically significant (p > 0.05) and was therefore not adjusted for in the present analysis. Most of the parturient women were primigravida in the interventional (64.0%) and control (68.0%) groups. In terms of antenatal exercise, most of the parturient women had not done any previous antenatal exercise regularly in both the control (80.0%) and interventional (76.0%) groups.

At baseline, the majority of parturient women in both groups experienced moderate pain (48.0% in the intervention group vs. 64.0% in the control group). As the intervention progressed, a shift toward lower pain levels was observed in the intervention group, with 40.0% reporting mild pain at post-test 1 and 36.0% at post-test 2 and 3. In contrast, the control group demonstrated a progressive increase in pain intensity, with 24.0% experiencing severe pain at post-test 2 and 60.0% at post-test 3. The mean VAS scores also reflected this trend, showing a steady decline in the intervention group and a marked increase in the control group across time points.

Table 1 shows the distribution of labor pain intensity across four time points among participants in both the intervention and control groups.

**Table 1:** Distribution of labor pain intensity at four time points in the intervention and control groups (N = 50) Data are presented as n (number of participants), f (frequency), and percentage (%). VAS: Visual Analogue Scale

Intensity of labor pain (VAS in mm)	Groups	N	Pre-test, f (%)	Post-test 1, f (%)	Post-test 2, f (%)	Post-test 3, f (%)
No pain (0-4)	Interventional	25	0 (0.0)	0 (0.0)	0 (0.0)	0 (0.0)
	Control	25	0 (0.0)	0 (0.0)	0 (0.0)	0 (0.0)
Mild pain (5-44)	Interventional	25	7 (28.0)	10 (40.0)	9 (36.0)	9 (36.0)
	Control	25	9 (36.0)	7 (28.0)	0 (0.0)	0 (0.0)
Moderate pain (45-74)	Interventional	25	12 (48.0)	11 (44.0)	12 (48.0)	14 (56.0)
	Control	25	16 (64.0)	18 (72.0)	19 (76.0)	10 (40.0)
Severe pain (75-100)	Interventional	25	6 (24.0)	4 (16.0)	4 (16.0)	2 (8.0)
	Control	25	0 (0.0)	0 (0.0)	6 (24.0)	15 (60.0)

A significant decrease in labor pain intensity among the intervention group parturient women, which was substantial in nature, was revealed by an RM-ANOVA test (F = 367 with 3 degrees of freedom, P-value < 0.001). Notably, a significant difference in the intensity of labor pain was observed between the two groups in post-test 2 (50 minutes after post-test 1) and post-test 3 (80 minutes after post-test 1 and 30 minutes after post-test 2), as determined by the ANOVA post hoc test (p = 0.005 and p = 0.001, respectively). Since both p-values are below 0.05, the differences are considered statistically significant. This provides evidence that warm compression was effective immediately following the second application and that the effects persisted even 30 minutes after the last administration. Therefore, following repeated application of warm compression, it can also be concluded that the effects are sustained and long-lasting.

Table 2 shows the comparison of labor pain intensity at different time points between the intervention and control groups using two-way RM-ANOVA with Bonferroni adjustment.

**Table 2:** Two-way repeated-measures analysis of variance (RM-ANOVA) showing the intensity of labor pain at different time points in the intervention and control groups (N = 50) Data are presented as N (number of participants) and mean ± SD (standard deviation). * indicates that the mean difference is statistically significant at the 0.05 level. Statistical significance was considered at p < 0.05.

Time	Intensity of labor pain in interventional group (in mm)		Intensity of labor pain in control group (in mm)		Bonferroni p-value	p-value
	N	Mean (SD)	N	Mean (SD)		
Pre-test (first contact with parturient women)	25	56.80 ± 20.25	25	48.01 ± 12.08	0.068	(<0.001)
Post-test 1 (20 minutes after pre-test)	25	50.08 ± 19.22	25	50.60 ± 10.44	0.906	
Post-test 2 (50 minutes after post-test 1)	25	50.64 ± 19.34	25	63.60 ± 10.95	0.005*	
Post-test 3 (80 minutes after post-test 1 and 30 minutes after post-test 2)	25	46.76 ± 19.29	25	76.32 ± 10.81	0.001*	

Figure 4 shows estimated marginal means of pain scores across time points for the intervention and control groups. The line diagram displays estimated marginal means of pain scores across time points for the control and experimental groups, derived from RM-ANOVA. The control group demonstrated a progressive increase in pain over time, whereas the experimental group showed a decrease in pain following warm compressions, with effects sustained up to 30 minutes after the second application.

**Figure 4:**
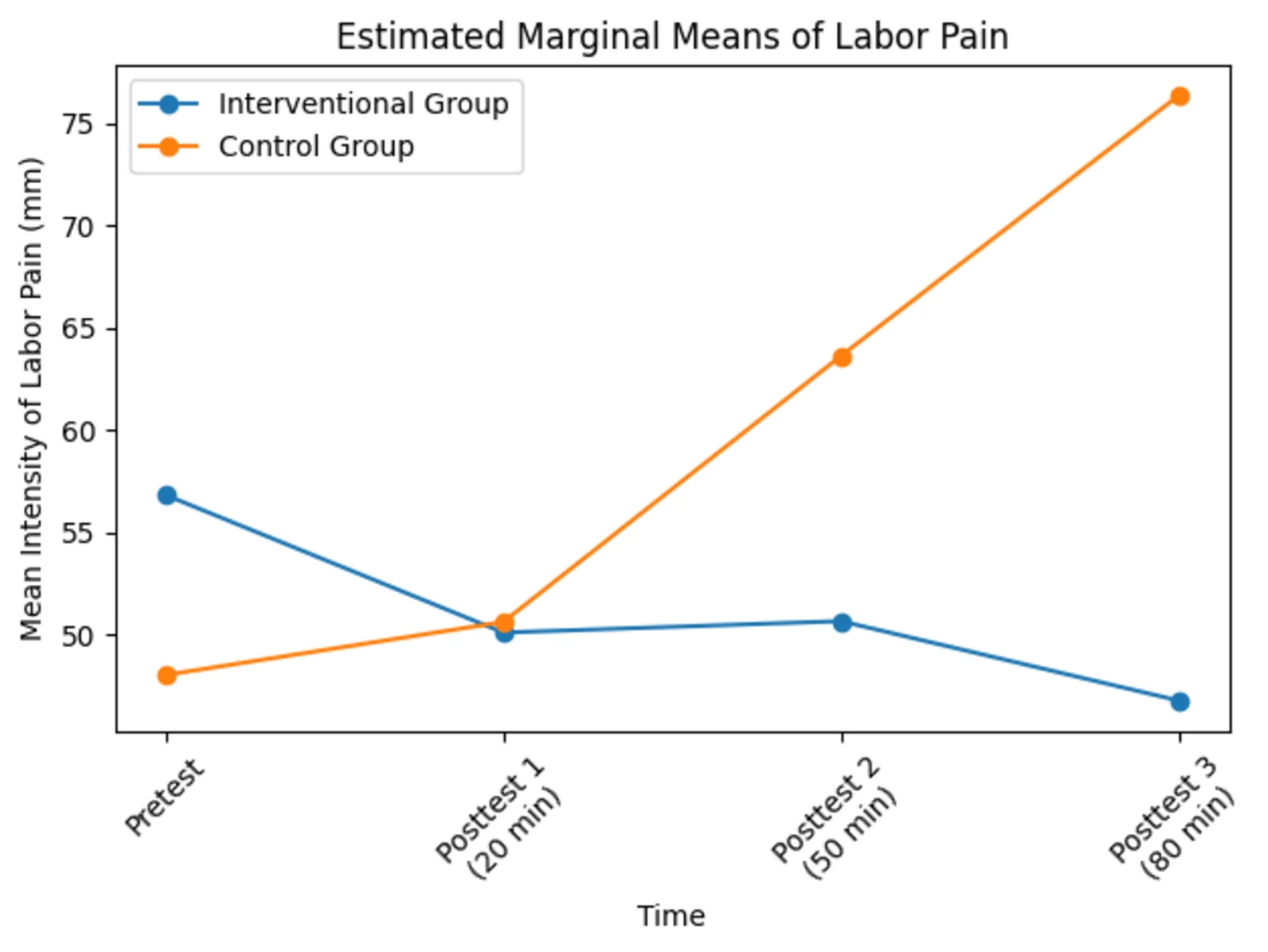
Line diagram displaying the intensity of labor pain in the intervention and control groups

In this study, no significant association was found between selected sociodemographic and clinical variable with intensity of labor pain.

## Discussion

The present study aimed to assess the effectiveness of warm compression at lumbo-sacral region on the intensity of labor pain during the first stage of labor. These findings are discussed in the context of existing literature. Study results revealed that immediately after the second warm compression and after 30 minutes of the second warm compression, the intensity of labor pain was significantly lower in the interventional group than in the control group (p < 0.05). These findings were supported by the study conducted in a district hospital, Ambala, India, which revealed that a significant difference was found between the control and interventional groups immediately after the first, second, and third warm compression, respectively (t = 3.20; p < 0.001; t = 4.45; p < 0.001; t = 6.18; p < 0.001) [15]. Similar findings were also noted in a quasi-interventional study conducted in Indonesia, where the study results showed that before warm compression, most of the mothers experienced very severe and severe levels of pain 53.3% and 43.3%, respectively whereas only 23.3% and 26.7% of the mothers experienced very severe and severe pain, respectively, after the warm compression. There was also a statistically significant reduction in pain level in the interventional group after warm compression (t = 18.330; p < 0.05) [18]. Another supporting study conducted in Turkey revealed that the application of massage and a hot pack reduced pain perception in the active phase at the 0.05 level of significance [19]. The above findings, with coexisting research, portray that warm compression was an effective method to reduce labor pain and enhance the comfort of the mother during labor.

No significant association was found in the present study between the intensity of labor pain and socio-demographic variables, which is supported by a similar study conducted at Pune, which revealed that there was no significant association found between the levels of pain and selected socio-demographic variables [13]. A study conducted by Jayanthi in the year 2011 also revealed that there was no significant association found between the level of labor pain and the selected demographic variables [20]. In contrast, a significant association was found between pain perceptions and socio-demographic variables (age, occupation) in the quantitative study conducted by Chithra et al. [21]. The study had several limitations, including the exclusion of parturient women with fewer than four observations and the subjective nature of labor pain, which may vary according to individual pain thresholds and emotional factors. The researchers also acknowledge the lack of blinding. These factors may have influenced the consistency and reliability of self-reported pain scores. Warm compression is a simple, low-cost intervention that can be incorporated into routine intrapartum care, particularly in low-resource settings. Safe implementation requires basic training of nurses and midwives on temperature monitoring and proper application techniques. Clean cotton cloths should be used in accordance with institutional infection prevention protocols, and the compress temperature should be monitored to ensure safety and consistency. Although the intervention requires some additional nursing time, it appears feasible within routine labour care. Future research should assess its acceptability and feasibility among women and healthcare providers across different maternity care settings. Involving larger and more heterogeneous populations across varied clinical environments is warranted to improve external validity. Comparative studies evaluating warm compression alongside other non-pharmacological labour pain interventions, as well as studies stratified by parity (primigravida versus multigravida), are also recommended to further establish its effectiveness and optimize labour pain management protocols.

## Conclusions

Warm compression is a simple, convenient, and non-pharmacological method that may help reduce pain intensity among parturient women during the first stage of labour. It may support maternal comfort and enable women to participate more actively in the birthing process while receiving continuous caregiver support. Therefore, warm compression may be considered a promising adjunctive strategy for labour pain management within routine nursing and midwifery care. However, further research, including larger multicentre studies, is required to establish its effectiveness across diverse populations and clinical settings and to determine its feasibility for wider implementation.
